# If You Pay, Will They Come? Evaluating the Impact of Subsidies on Cessation Outcomes in the Walk or Run to Quit Program

**DOI:** 10.1155/2022/7929060

**Published:** 2022-09-09

**Authors:** Kelly B. Wunderlich, Daniel Do, Hannah Martin, Carly S. Priebe, Guy E. J. Faulkner

**Affiliations:** School of Kinesiology, University of British Columbia, Vancouver, BC, Canada V6T 1Z1

## Abstract

**Introduction:**

Exercise interventions may assist smoking cessation attempts. One such publicly available 10-week program, Walk or Run to Quit (WRTQ), demonstrated success in smoking cessation and physical activity (PA) outcomes. However, initial WRTQ participants (2016-2017) were fairly homogenous in their demographic profile. To increase diversity, subsidies for participation were offered in 2018. This study assessed how the subsidies affected participant demographics, running frequency, smoking cessation, intention to quit, and program attendance and completion.

**Methods:**

The $70 registration fee was subsidized for 41% of participants in 2018. A pre-postdesign was used, with participants completing surveys on their demographics and smoking and physical activity behaviours. Descriptive statistics compared the year subsidies were available (2018) and unsubsidized years (2016-2017) and subsidized and unsubsidized participants' data from 2018.

**Results:**

The 2018 participants had lower average attendance and program completion rates compared to 2016-2017 and no statistically significant differences in demographics or smoking cessation and PA outcomes. There were no differences in smoking cessation, run frequency, or demographic variables between the subsidized and unsubsidized participants in 2018.

**Conclusions:**

Offering subsidies did not diversify the participant profile. Subsidies did not have a negative impact on attendance nor primary outcomes. Subsidies may not have addressed barriers that prevented a more diverse sample from participating in WRTQ, such as program location, timing, and design. Equitable access to smoking cessation programs remains essential. As subsidies may play a role in reducing financial barriers disproportionately faced by marginalized groups, the implementation of, and recruitment for, such subsidized programs requires further investigation.

## 1. Introduction

Tobacco use is the most preventable cause of death and disease [1]. Most people who smoke want to quit. For example, one US study found 68% of smokers want to quit [2]; however, most quit attempts without support are unsuccessful. It is recognised that interventions using a combination of pharmacotherapy and behavioural support are most effective [3]. While multipronged approaches appear to yield initial quit success, further investigation into behavioural modification programs that can sustain successful quit outcomes and prevent relapse is warranted [4].

Among other existing strategies, exercise may serve as a promising smoking cessation approach given its broad and beneficial proposed effects including substantial evidence that acute bouts of exercise decrease nicotine withdrawal symptoms and alleviate mood disturbance [5]. There is also evidence that exercise may suppress appetite which may be highly significant for those looking to avoid weight gain during a cessation attempt [6]. The reductions of anxiety sensitivity and dysphoria, which can be accomplished through exercise, are two independent mechanisms that explain cessation success [7]. However, despite plausible mechanisms through which exercise may assist smoking cessation, there is currently modest evidence that incorporating exercise into smoking cessation attempts improves abstinence compared to traditional treatment alone [8]. Despite this, researchers have suggested that the hypothetically intersecting mechanisms provide sufficient motive to continue developing and testing exercise-based smoking cessation programs [9].

One such program is the Walk or Run to Quit (WRTQ) physical activity-based smoking cessation program. WRTQ is a Canadian program formed through a multisectoral partnership between an industry (Running Room), nonprofit (Canadian Cancer Society), and academic (UBC) partnership combining other smoking cessation strategies with an overarching exercise intervention in an effort to decrease smoking prevalence and increase overall physical activity and running frequency of participants [10]. Overall, the 10-week program exhibited potential as a scalable intervention that can simultaneously target both smoking cessation and physical activity (see [11, 12]).

After the first year of the program, 90.8% of the participants who completed the in-person clinics reported reducing their smoking because of the program. Over 50% of the program completers did not smoke a cigarette within the last week of the program as confirmed by carbon monoxide testing. Despite these benefits, there was some concern about the homogeneity of the participants in recruiting white, college educated individuals from higher socioeconomic backgrounds [11]. The majority of participants ranged from 40 to 59 years old, and 71% were female. Ninety-four per cent of participants identified as white, with 65.6% fully employed and 70% owning their home. In terms of participant education level, 68% had received at least a college education [11]. This does not reflect the broader demographic profile of smokers in Canada who tend to be male and older and report lower income [13].

One barrier to broader representation may have been the cost of participation (a $70 registration fee). In 2018, some participants had their registration fee subsidized so that they did not have to pay to join the program. This subsidy implementation is of interest as price-based policies, including the use of subsidies, can directly address financial cost as a barrier. In Canada, subsidization policies were associated with modest increases in the use of nicotine replacement therapy (NRT) use and quit success [14]. Previous research has explored the role of incentives on cessation outcomes [15, 16] and recruitment [17] and has found them to be effective. As defined by the search terms used by Sigmon and Patrick, incentives in this context are often contingent on cessation outcomes. This is in contrast to subsidies in programs like WRTQ where the cost of the program is waived for some participants. Research on the impact of subsidization on exercise programs and/or multicomponent cessation programs including its effect on the demographic profile of participants is sparse. Therefore, this current study addressed two objectives: the first was to identify whether subsidies were successful in diversifying participant demographics in the program, and the second was to identify if there were any associations between subsidization and program attendance, completion rates, running frequency, and smoking cessation. It was hypothesized that subsidies would be effective in diversifying the participant demographics. No a priori hypotheses were set regarding program outcomes.

## 2. Methods

### 2.1. Participants

Participants were Canadian adults (*N* = 745) registered for WRTQ clinics at Running Room stores across Canada (see [12] for further details). Participants were eligible to register if they were at least age of majority in the province within which they registered (e.g., 18 years in Alberta), a Canadian citizen, a current smoker or commercial tobacco user, or someone who quit within the last three months and had smoked at least 100 cigarettes in their life. Family and friends who provided social support to participants could also register as “buddies.” All registered participants were invited to take part in the evaluation.

WRTQ in-person clinics were held at various Running Room stores across Canada, with a registration fee of $70 CAD per person. This registration fee covered access to the program which included a 10-week smoking cessation and running curriculum led by a coach. Nicotine replacement therapy was available outside programs but was accessible and free/included for all participants as were provincial quitline supports (e.g., https://quitnow.ca).

During the third year of the program's implementation, full subsidies were incorporated and distributed by health care professionals to a portion of potential participants with the intention of increasing the diversity of participation [12]. This was accomplished by providing coupon codes to health care practitioners and the national quit smoking line to share with patients and callers eligible for the program. They were instructed to identify smokers who felt that the cost of the program was a barrier. Staff from the Canadian Cancer Society distributed the coupon codes at fairs, at community events, and to individuals outside on smoke breaks. Indigenous community leaders and rural health centres were also given the coupon codes for distribution.

During the first two years (2016 and 2017; *n* = 384), there was a $70 CAD registration fee. In the third year (2018; *n* = 331), 58.9% paid the $70 fee while the remaining (41.1%) received a subsidy. For these analyses, participants were excluded if they were a nonsmoking buddy (*n* = 30) since this would artificially decrease the 7-day PPA scores.

### 2.2. Research Design and Procedure

A pre-postdesign with paper and pen surveys were used to collect self-report data on physical activity and smoking behaviours. These surveys were completed at weeks 1 and 10. The week 1 survey also include demographic information (i.e., sex, education, and home ownership). Device-measured carbon monoxide (CO) was collected using a coVita piCO+ Smokerlyzer® device at weeks 1 and 10. Coaches completed a log each week that tracked participants' attendance.

### 2.3. Measures

*Attendance*. Program attendance was collected (1-10 sessions).

*Completion status*. Completion of the program was defined as both attending and completing postprogram measures at week 10.

*Smoking status*. Smoking status was represented by 7-day point prevalence abstinence (PPA), which assesses whether participants smoked in the past week. A survey question (have you smoked, even a puff, in the last 7 days?) and CO scores were used to determine 7-day PPA. Meeting 7-day PPA was defined as not having a puff in the last 7 days and having a CO level of <10 ppm at the week 10 assessment.

*Run frequency*. Physical activity at week 10 was assessed with the item “How many times/week do you currently run (for at least 10 minutes at a time), if at all?”

*Intention to quit smoking*. Intention to quit in the next 30 days was measured at week 1 using a single item with dichotomous yes/no response options. “Are you seriously considering quitting within the next 30 days?”

*Participant demographics*. Demographics reported at baseline included sex (male/female), race/ethnicity (categorized as white/all other responses for the purposes of analyses), age, highest level of education completed (secondary school or less/postsecondary education (college, university, graduate school)), home ownership (own/rent), and employment status (employed full-time/all other responses (employed part-time, student full-time, student part-time, self-employed, at home with children, without paid employment, and not applicable)). Socioeconomic status “(or sometimes socioeconomic position) refers to standing in the stratification system and is usually measured by education, occupation, employment, income, and wealth.” ([18], p. 351). There was no direct measurement of income but home ownership, employment status, and education were used as indirect measures.

### 2.4. Data Analyses

Descriptive statistics, including chi-square (*χ*^2^) tests, depicted participant demographic information as well as completion and smoking status. Independent samples *t*-tests assessed the differences in attendance and run frequency. These tests were used to compare unsubsidized and subsidized participants in 2018 and whether there were any differences between participants who took part pre- (2016-2017) and postsubsidization (2018). An intent-to-treat (ITT) approach was used for 7-day PPA and run frequency. If participants did not have data for both survey and CO criteria (i.e., they did not complete the program), it was assumed that they were still smoking and did not meet 7-day PPA. Participants missing week 10 running frequency data were assumed to have the same running frequency as baseline before starting the program.

## 3. Results

### 3.1. Participant Demographic

An overview of the demographic profile of participants in all years, pre- and postsubsidization, and for participants who were and were not subsidized in 2018 is provided in Table 1. Results from the statistical tests are also included. Overall, the sample was primarily female (74.9%), was white (93.5%), had completed postsecondary education (78.3%), was employed full time (65.8%), and was 40-59 years old (64.2%).

### 3.2. Outcomes

#### 3.2.1. Pre- and Postsubsidization

Pre- and postsubsidization results were compared to explore whether the partially subsidized year had a comparable demographic profile and program outcomes to years without subsidies. There were statistically significant differences for program completion status (presubsidization = 47.4%; postsubsidization = 37.5%; *χ*^2^ (1, *N* = 715) = 7.2, *p* < .01). There were also statistically significant differences for attendance, with participants in the presubsidization years attending more sessions (*M* = 5.5, SD = 3.0) than those in the postsubsidization year (*M* = 4.9, SD = 3.0; *t*(652) = −2.8, *p* < .01). There were no statistically significant differences (*p*′s > .05) for smoking status, sex, identifying as white, education, age, home ownership, employment status, run frequency, or intention to quit smoking.

#### 3.2.2. Subsidized and Unsubsidized Participants

There were no statistically significant differences (*p*′s > .05) for any demographic or outcome variables when comparing participants who were subsidized and unsubsidized in 2018.

## 4. Discussion

WRTQ was a nationwide Canadian program consisting of group-based clinics that utilized varying strategies to target smoking cessation and physical activity. Despite results supporting the program's utility in decreasing smoking status and increasing physical activity with moderate-to-strong effects on indicators of participants' health behaviours, the homogenous and unrepresentative participant demographic profile was recognized as an area for improvement [12]. This recognition subsequently led to the incorporation of full program subsidization for select participants to diversify participation (i.e., patients who were identified by a health care practitioner as someone for whom cost was a barrier to participation). Our results suggest that subsidies were unsuccessful in this regard. There were differences in the proportion of participants who completed the program and average number of sessions attended when comparing the year when subsidies were offered and the years that they were not. This might have been a result of the national scale-up of the program between years 2 and 3 and variability in intervention fidelity. On a positive note, subsidies did not appear to undermine participants' motivation and success as there were no statistically significant differences in program attendance, drop-out, running frequency, intention to quit, and smoking cessation between those who received the subsidies and those who did not within the same year.

Our findings do not rule out a potential role of subsidies in future programs like WRTQ. Given no difference in the demographic profile of those receiving or not receiving subsidies, it may be that the mechanism for distributing subsidies was ineffective. As a secondary analysis, tracking of the subsidy distribution process was not conducted and as a result, we are unable to comment on this process. Future programs with subsidy components could be more deliberate in assessing the effects of subsidies by also exploring the experiences of those that received subsidies. It may be that the subsidies were necessary for some individuals where cost was a real barrier to participation. Only collecting proxy measures of income (home ownership, employment status, and education) is a limitation of the analysis. Current research regarding the effects of subsidies on physical activity participation remains limited, and future research is warranted regarding how subsidies are effectively delivered to alleviate financial barriers.

Another consideration is that the nature of the WRTQ program was potentially not attractive to a more diverse group of participants for two reasons. First, as a multisectoral health partnership between a health charity, Canadian Cancer Society and an industry organization, Running Room, the programs were provided at the organization's Running Room stores. These stores are located in high-density, urban settings. Such settings may not have been accessible to many individuals who could have benefited from the program. Future iterations of the WRTQ program should implement broader outreach and move “clinics to the communities.”

Second, the connotations associated with leisure time physical activity (such as running or walking) may also be a barrier to participation for a broader demographic of participants. Adults with lower SES may be more physically active at work and less active during their leisure time [19, 20]. This occupational physical activity may be a barrier to leisure-time physical activity, making a program like WRTQ less appealing. Examples of barriers to leisure time physical activity for low SES adults include poor urban planning that may deter activity in their own neighbourhoods, program times that do not account for work schedules and availability of childcare, not having clothes that feel comfortable while being active in public or outside, and family or friends considering making time for physical activity to be selfish [21]. Additionally, financial barriers can also extend beyond the registration fee, such as the cost of childcare and transportation that are required to participate [21]. Simply, providing free registration does not remove all financial barriers to participation. The design of the WRTQ program, combined with its location and timing, may have been unappealing to a more diverse audience of Canadians looking for smoking cessation support.

There are a number of strengths and limitations associated with this particular program evaluation. The study is limited in that it could not account for extraneous variables that may have influenced subsidy uptake. Income was not directly measured nor was subsidy distribution adequately assessed. Strengths of this study include its national scale and reach, the use of objective measures for smoking cessation, and the ability to compare program demographics both over different program years with and without subsidies and across a single program with some participants receiving the subsidy intervention.

## 5. Conclusion

Integrating physical activity into smoking cessation interventions may have synergistic health benefits through multiple health behaviour change. Irrespective of cessation outcomes, increasing physical activity may confer some harm reduction benefits [22]. The impact of Walk or Run to Quit on cessation outcomes was comparable to other multicomponent interventions [12] while also increasing physical activity among those who completed the program. Ensuring equitable access to programs like Walk or Run to Quit should be a priority given the intersections between racial/ethnic and socioeconomic disparities in both smoking [23, 24] and physical activity [25, 26]. In this case, subsidization did not expand the relatively narrow demographic profile of participants. Future iterations will need more careful consideration of the recruitment design and cocreate strategies to overcome barriers to broader participation [27]. This may require reconceptualizing what subsidies are targeting beyond registration cost and the content of the program itself.

## Figures and Tables

**Table 1:** Overview of demographic profile for all years, and differences between pre- (2016-2017) and postsubsidization (2018) and subsidized and unsubsidized participants (2018).

		Presubsidies vs. postsubsidies						Subsidized vs. unsubsidized					
	Overall (2016-2018)	Presubsidies	Postsubsidies	df	*N*	*χ* ^2^ statistic	*p* value	Subsidized	Unsubsidized	df	*N*	*χ* ^2^ statistic	*p* value
% completed	42.8	47.4	37.5	1	715	7.17	<.01	37.5	37.4	1	331	.00	.99
% meet 7-day PPA	21.0	22.4	19.3	1	715	1.00	.32	20.6	18.5	1	331	.23	.63
% female	74.9	72.3	77.9	1	700	2.88	.09	76.3	79.0	1	321	.34	.56
% white	93.5	93.6	93.4	1	694	.02	.89	94.7	92.5	1	317	.59	.44
% completed postsecondary education	78.3	75.7	81.4	1	701	3.26	.07	81.5	81.3	1	322	.00	.96
% own home	71.7	72.7	70.4	1	695	.46	.50	72.6	68.8	1	321	.54	.46
% employed full-time	65.8	65.6	65.9	1	701	0.01	.93	65.2	66.5	1	323	.06	.81
Age (years)													
% younger adults (18-39)	25.2	25.8	24.5	2	702	.34	.84	21.5	26.7	2	322	5.53	.06
% middle age adults (40-59)	64.2	64.2	64.3					71.1	59.4				
% older adults (60+)	10.5	10.0	11.2					7.4	13.9				
% intending to quit	90.4	91.6	88.9	1	656	1.42	.23	92.7	86.2	1	297	3.06	.08

				df		*t*-statistic	*p* value			df		*t-*statistic	*p* value
Mean attendance	5.22	5.52	4.86	652		-2.80	<.01	4.72	4.96	300		-0.68	.50
Run frequency	1.12	1.16	1.06	693		-0.88	.38	1.21	0.95	314		.12	.12

## Data Availability

In the interest of guarding the privacy and confidentiality of the WRTQ participants and as necessitated by our ethics, the raw data will not be shared.
